# *LubriShield*^TM^—A permanent urinary catheter coating that prevents uropathogen biofilm formation *in vitro* independent of host protein conditioning

**DOI:** 10.1371/journal.pone.0328167

**Published:** 2025-07-10

**Authors:** Ana I. Romero, Serhiy Surkov, Per Wirsén, Graeme Brookes, Linda Bergström, Jan Tejbrant, Elena Dhamo, Sandra Wilks, Catherine Bryant, Jan Andersson

**Affiliations:** 1 CytaCoat AB, Solna, Sweden; 2 School of Biological Sciences, University of Southampton, Southampton, United Kingdom; 3 Department of Medicine, Karolinska Institutet, Stockholm, Sweden; Universidad Autonoma de Chihuahua, MEXICO

## Abstract

Catheter-associated urinary tract infection is one of the most common healthcare-associated infections, with biofilm formation playing a key role in its pathogenesis. Indwelling medical devices introduce ideal pathways inside the body for pathogens and feature surfaces conducive to biofilm development, often leading to severe clinical infections recalcitrant to antimicrobials. When bacteria and fungi switch to biofilm mode of growth, they produce a matrix in the form of extracellular polymeric substances (EPS). This creates a unique environment for growing virulent colonisers and persisting cells while forming a shielding barrier against immune system attacks, antimicrobial agents and mechanical removal by fluid shear forces. To address this challenge, LubriShield^TM^ – a novel permanent coating – was invented and evenly applied to both internal and external surfaces of indwelling urinary Foley catheters. Without releasing active substances, it effectively prevented pathogens from producing biofilm. The superhydrophilic coating, incorporating a proprietary anti-fouling ligand, significantly inhibited colonising uropathogens from forming biofilm for up to 14 days in artificial urine medium without microbial killing (up to 99% reduction, *P < 0.001*). In a glass bladder flow model, LubriShield^TM^ still significantly reduced biofilm formation by 83*% (P < 0.0001)*. Importantly, LubriShield™ maintained its antibiofilm efficacy even after conditioning with fibrinogen, a host-derived protein known to promote bacterial attachment (*P = 0.007*). RNA-seq analysis revealed significant downregulation of genes associated with microbial EPS formation on the coated surfaces. Additionally, microorganisms adhering to LubriShield^TM^ coated catheters showed a 78% increased susceptibility to antibiotics compared to those on uncoated catheters (*P = 0.004*).

## Introduction

Among urinary tract infections (UTIs) acquired in the care of patients, approximately 75% are associated with the use of urinary catheters [1]. Catheter-associated urinary tract infection (CAUTI) is usually caused by biofilms formed by microorganisms originating from the subject’s own colonic and perineal microflora (*Escherichia* *coli, Klebsiella pneumoniae, Proteus mirabilis, Pseudomonas aeruginosa, Enterococci*), but can also come from the hands of healthcare personnel or the patient’s skin during catheter insertion and manipulation with the collection system (*Staphylococcus epidermidis* or S*taphylococcus aureus*). CAUTIs may lead to potentially serious consequences, including frequent febrile episodes, acute and chronic pyelonephritis, bacteraemia with urosepsis and septic shock, catheter obstruction and renal and/or bladder stone formation, and even bladder cancer with prolonged use [2–4].

Catheters provide an ideal surface on which bacteria can adhere and develop biofilms [5,6]. The catheter-associated biofilm is a three-dimensional structure triggered by the microorganism’s surface sensing and initial adhesion, followed by colonisation and the production of a slimy matrix composed of extracellular polymeric substances (EPS) [7]. It represents a predominant form of microbial life with highly organised communities of microorganisms, including bacteria, fungi, and, at times, mixed-species populations. Within a biofilm, distinct population zones emerge as a result of environmental gradients. Persistent cells often reside near the base of the biofilm in anaerobic regions, where nitrate respiration dominates in the absence of oxygen. In contrast, metabolically active, oxygen-respiring cells occupy the biofilm’s outer layers, poised for dispersal to new surfaces [8]. Only approximately 10% of the biofilm mass represents microbial cells, while most of it consists of hydrated EPS. Polysaccharides, proteins and extracellular DNA (eDNA) are the main components of the EPS, but it can also contain varying amounts of membrane vesicles, lipids, amyloids, cellulose and appendages, such as cell-wall-anchored proteins, pili, fimbriae, and flagella. These components not only provide structural stability to the biofilm but can also contribute to inflammatory conditions [9].

Catheter-associated biofilms provide uropathogens with many survival advantages, including resistance to the shear stress of the urine flow, resistance to phagocytosis, and resistance to antimicrobial agents [10–12]. In addition, some common uropathogens, such as *Proteus species*, *P. aeruginosa*, and *K. pneumoniae*, produce an active urease that can hydrolyse urea in the urine to free ammonia [13]. This results in a rapid increase in local pH leading to precipitation of minerals, such as hydroxyapatite and/or struvite. Encrustations of this kind are seen typically on the inner lumen of the catheter and can build up to block the catheter flow completely [14]. Despite continued efforts to produce effective catheter materials and coatings that resist biofilm development, the problems of CAUTI and blockage prevail. Coatings that kill bacteria by release only have limited effect in the immediate surroundings and exhaust after a while. To date, bactericidal and bacteriostatic materials, such as silver-coated or antibiotic-impregnated coatings, have not been found to improve clinical outcomes significantly over long-term use [10,15,16]. Other attempts with antibacterial topological coatings that kill bacteria upon adhesion often get compromised by the accumulation of dead cells and associated debris on the surfaces [17]. These coatings do not prevent biofilm formation, but promote antimicrobial resistance, generate endotoxin release from dying bacteria and are potentially toxic to the human body. Thus, there is an unmet clinical need for effective long-lasting, non-release protection against biofilm formation on indwelling catheters. By understanding how microorganisms sense a surface and what signals make them switch into a virulent lifestyle and biofilm mode of growth, more efficient coatings can be developed to protect against catheter-induced infections.

**Fig 1:**
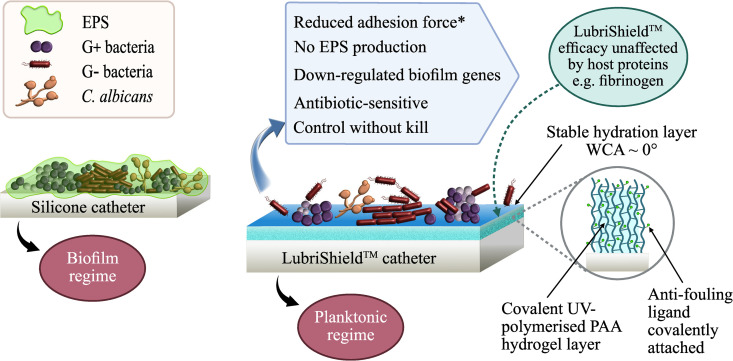
Schematic representation of microbial colonisation on an uncoated silicone catheter versus a LubriShield™ coated catheter. The uncoated silicone catheter (left) facilitates biofilm formation by Gram-negative and Gram-positive bacteria and *Candida albicans*, embedded within an extracellular polymeric substance (EPS) matrix (green). In contrast, the LubriShield™-coated catheter (right) features a covalently bound polyacrylic acid (PAA) hydrogel layer functionalised with anti-fouling ligands. This creates a stable hydration barrier with a water contact angle (WCA) near 0°, reducing adhesion forces* and inhibiting microbial transition to a biofilm mode. Microorganisms remain antibiotic-sensitive, while biofilm gene expression and EPS production are down-regulated. Importantly, this mechanism enables control without kill, thereby minimising resistance development. The coating retains its efficacy even in the presence of host proteins, such as fibrinogen. *Reduced adhesion force is a literature-supported theoretical mechanism [18–21] proposed to explain observed biofilm inhibition, and has not yet been directly confirmed experimentally in this work.

Here we present LubriShield^TM^, a permanent non-release superhydrophilic coating consisting of a covalently bound polyacrylic-acid hydrogel functionalised with a proprietary anti-fouling ligand. This coating effectively inhibited microbial biofilm formation on catheter surfaces under both static and dynamic culture conditions. The LubriShield^TM^ coated CytaCoat Foley catheter was biocompatible, non-toxic and exhibited up to 28-fold friction reduction when wetted, compared to uncoated silicone Foley catheters. Coated catheters challenged with common uropathogens (*E. coli*, *K. pneumoniae*, *P. mirabilis*, *P. aeruginosa*, *Enterococcus faecalis*, *S. epidermidis*, *S. aureus*, and *Candida albicans*) for up to 14 days in artificial urine showed significant biofilm inhibition, as determined by morphological, biochemical and genetic analyses. We also examined fibrinogen adsorption, a clinically relevant biofilm-promoting factor. To illustrate this mechanism, Fig 1 schematically represents LubriShield™‘s non-biocidal microbial control strategy. The coating promoted a planktonic regime by reducing microbial adhesion forces and down-regulating biofilm-associated genes, rather than exerting bactericidal pressure. This mechanism thus represents a promising strategy for durable, biocompatible prevention of catheter-associated biofilms, potentially reducing infection risk. The ability of bacteria to sense and respond to adhesion forces (mechanosensing) may underpin this mechanism, where reduced surface adhesion impairs biofilm formation [18–21].

## Materials and methods

### LubriShield^TM^ coating

#### Medical devices.

The coating was applied on a CE-marked medical grade silicone Foley catheter certified according to ISO 13485 (Sterimed Group). The uncoated silicone Foley catheter was used for all comparisons.

#### UV-induced polymerization.

The grafting of silicone catheters was initiated via a UV-induced polymerization process. Prior to grafting, the catheters were dipped in a benzophenone (photo-initiator) ethanol solution. After drying, the catheters were immersed in an aqueous grafting solution containing acrylic acid under UV irradiation. Washing in deionised water and then ethanol was performed after the grafting process. The uniformity of the grafted surfaces of the silicone catheters was analysed by staining in an aqueous solution containing methanol crystal violet (4%). After thorough washing in deionised water the colouring of the coated silicone catheters was compared with a stained ungrafted silicone catheter (S1 Fig).

#### Coupling of the proprietary antifouling ligand.

The anti-fouling ligand, “2-(pyridyldithio)ethylamine (PDEA)”, was attached to the carboxylated surface by chemical coupling reactions essentially as reported in the literature [22]. In short, the carboxylic groups in the grafted layer were activated with an aqueous solution of 1-ethyl-3-(3-dimethylaminopropyl) carbodiimide (EDC) and *N*-hydroxysuccinimide (NHS) and then reacted with PDEA. Intermittent washing in deionised water was performed for each reaction. Chemical characterisation of the coated catheter, performed in accordance with ISO 10993−18 (AMD-1:2022), revealed no detectable release of coating components or impurities for volatile, semi-volatile, non-volatile organic compounds, and elemental impurities (S1 Table).

#### Water Contact Angle visualisation and measurement.

Measurement of the water contact angle, WCA, by sessile drop imaging analysis was performed by Optas LTD (UK), an independent expert house. The samples were pre-wetted in HPLC Grade water before measuring the contact angle at the water – air – film surface interface with a tracking oscillating drop tensiometer (Teclis Scientific).

#### Friction assessment.

Measurements of the dynamic coefficient of friction were performed by Optas Ltd. (UK), according to ISO 8295. Catheter samples were pre-wetted in HPLC-grade water prior to applying a sled weight of 90 g. Following a three-second delay, measurements were carried out at a speed of 24 mm/minute over a distance of 20 mm. Importantly, rigorous handling tests confirmed that upon drying, catheter surfaces remained non-sticky, effectively preventing unintended adherence to tissues during insertion. Upon rewetting, samples immediately regained their initial low dynamic coefficient of friction, demonstrating stable and reversible lubricious properties of the coating.

### Microbiology

#### Materials.

The following materials were used for the microbiological study work: Luria broth and Luria agar plates (Karolinska Hospital Substrate unit, Stockholm, Sweden), phosphate buffered saline (PBS, Karolinska Hospital Substrate unit, Stockholm, Sweden), crystal violet (CAS 548-62-9, Acros Organics, USA), EbbaBiolight 680 (Ebba Biotech, Sweden), concanavalin A (Alexa Fluor® 594 conjugate, Thermo Fischer Scientific, USA), FilmTracer™ SYPRO™ Ruby Biofilm Matrix Stain (Thermo Fischer Scientific, USA), colistin sulphate (CAS 1264-72-8, MedChemExpress, USA), vancomycin hydrochloride (CAS 1404-93-9, MedChemExpress, USA). Artificial urine medium (AUM) was freshly prepared according to Brooks T. et al. 1997 every day in the lab, pH adjusted to 6.5 and filter sterilised before use [23]. Chemicals used for AUM preparation are listed in S2 Table.

#### Strains used.

A set of microorganisms, representing clinical isolates of Gram-positive and Gram-negative bacteria species, as well as *Candida albicans*, was used (S3 Table) and obtained from Department of microbiology, Karolinska University hospital.

#### Culturable count.

Culturable count was done by counting microorganism colony forming units. For bacteria in liquid medium, a serial 10x dilution in 1 mL of PBS was done. 100 µL of resulting microorganism suspensions was placed on Luria agar plates, spread with sterile glass beads and incubated at 37°C overnight. Colonies were counted and CFU/mL in original culture calculated. For surface-attached bacteria, catheter pieces were placed in 1.5 mL of PBS in a 2-mL microcentrifuge tube and sonicated with Cell Disruptor (AOSENR, China) 30 seconds sonication/ 10 minutes cooling/ 30 seconds sonication to remove attached bacteria and disrupt biofilm. Resulting suspension was used for serial dilutions and colony counting as described above.

#### Static biofilm growth test.

The test was performed on 2-cm catheter segments placed into 2 mL-microcentrifuge tubes with only shaking creating shear force necessary for the biofilm formation. 1 mL of freshly made AUM containing resuspended 10^9^ CFU/mL for bacteria or 10^8^ CFU/mL for *Candida* was added. Tubes were incubated on a rotary shaker (90 rpm) at 37°C and media was changed daily. After seven days or more, the pieces were removed, gently rinsed and analysed.

#### Dynamic biofilm growth test with flow model.

The test was performed on 7-cm catheter sections placed in line with a peristaltic pump for a continuous medium flow. *P. mirabilis,* 10^9^ CFU/mL, in freshly made AUM was seeded inside the catheters for 30 minutes before connecting to the system and initiating flow. Cultures were kept at 37°C and continuously supplied with fresh medium at a rate of 1 mL/minute. After 3 days, the sections were removed, gently rinsed and analysed.

#### Dynamic biofilm growth test with glass bladder flow model.

The model consists of a glass vessel maintained at 37°C by a water jacket (Fig 3B, left panel). The model was sterilised by autoclaving and then a catheter was inserted aseptically into the vessel through a section of silicone tubing attached to a glass outlet at the base. The catheter balloon was inflated with 10 mL of water, securing the catheter in position and sealing the outlet from the bladder. The catheter was then attached to a drainage tube and a reservoir bag located below the level of the glass vessel. Sterile AUM was supplied to the bladder via a peristaltic pump. In this way a residual volume of urine collected in the bladder below the level of the catheter eyehole. As AUM was supplied to the model, the overflow drains through the catheter to the collecting bag. Six models with three LubriShield^TM^ coated catheters and three uncoated catheters were set up to study the biofilm formation of *P. aeruginosa*, responsible for about 10% of all CAUTI [24]. An inoculum of 100 µL of 10^9^ CFU/mL of *P. aeruginosa* in AUM was added to the residual volume in the vessel for 1 h to establish itself before the supply of AUM, at a rate of 1.5 mL/min, was switched on. After five days the supply of AUM to the bladder was turned off, the balloon was deflated, and the catheter was removed through the base of the model.

**Fig 2:**
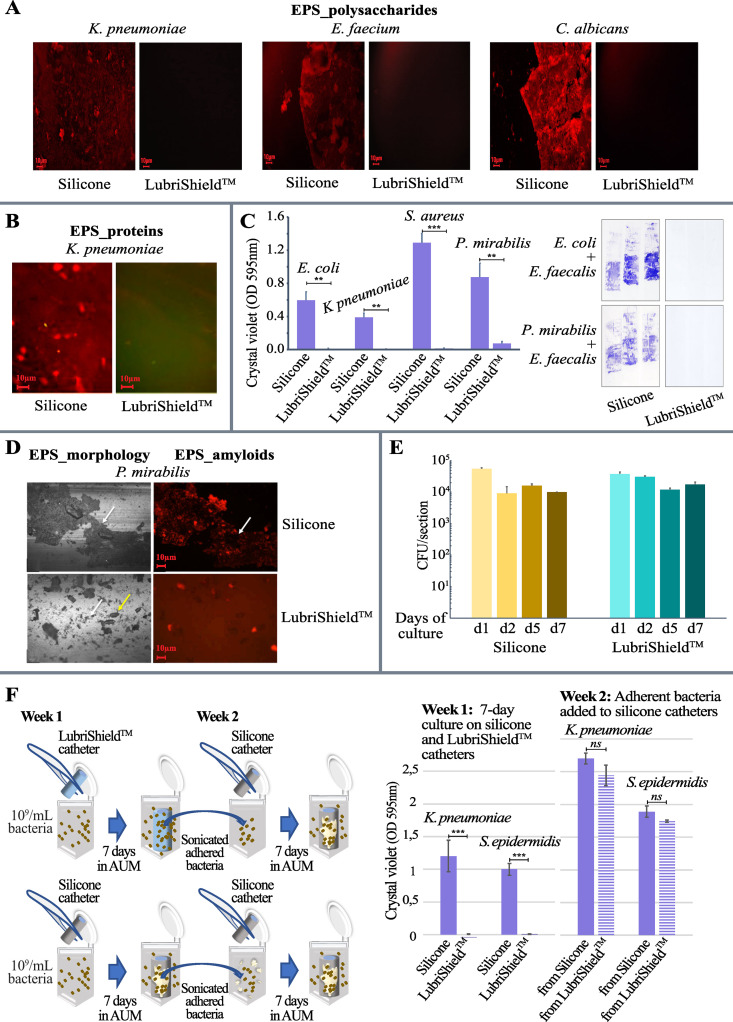
Assessment of up to 14 days biofilm cultures in artificial urine of different uropathogenic strains on uncoated and LubriShield^TM^ coated silicone catheters. (A) 7-day cultures in artificial urine under static conditions show significantly reduced production of polysaccharides by *K. pneumoniae*, *E. faecalis* or *C. albicans* on LubriShield^TM^ catheter surfaces. Microscopic images of concanavalin A fluorescent staining (Alexa Fluor™ 594 conjugate) comparing control silicone- versus LubriShield^TM^ catheter surfaces (repeated three times). (B) Microscopic images of SYPRO Ruby fluorescent staining (FilmTracer^TM^ SYPRO® Ruby) of proteins by *K. pneumoniae* comparing control silicone- versus LubriShield^TM^ catheter surfaces (repeated three times). (C) Quantification of polysaccharides and proteins in the EPS by crystal violet shows no biofilm after 14 days of static culture with different uropathogens (Student’s t test: n = 3, ***: *P < 0.001*, **: *P = 0.005* (*E. coli*), *P = 0.002* (*K. pneumoniae*), *P = 0.009* (*P. mirabilis*)). The right panel shows crystal violet staining on adhesive tape transferred from 14 day mixed-species culture on control silicone- versus LubriShield^TM^ catheter surfaces. (D) Images of parts of catheter surfaces after 5 days of incubation in AUM showing EDIC/EF images in the left panels and EbbaBiolight 680 staining of EPS in the right panels. Magnification = X100, White arrows = biofilm, Yellow arrows = crystal formation. (E) Colony forming units (CFU) per section of *P. mirabilis* bacteria over time incubated in AUM comparing silicone catheter surface to LubriShield^TM^ surface (n = 3). (F) Schematic representation of the conducted experiment assessing biofilm-forming abilities of bacteria on LubriShield^TM^ surface (left). Crystal violet counts showing biofilm formation of the *P. mirabilis* residing on LubriShield^TM^ catheter and on silicone catheter surface (week 1) or removed from the corresponding surface and re-cultured on uncoated silicone (week 2 (n = 3, ***: *P < 0.001*, ns: nonsignificant, Student’s t test).

#### Fibrinogen (Fg) preconditioning and biofilm assay.

Sterile catheter segments (2 cm) were incubated in human Fg solution (1 mg/mL in PBS, Merck) at 37°C for 1 h under static conditions in sterile glass tubes. After incubation, the Fg solution was gently aspirated, discarded, and catheter pieces were washed three times with PBS to remove non-adherent fibrinogen. Subsequently, catheter pieces were incubated for 7 days at 37°C under static conditions with *K. pneumoniae*, 10^9^ CFU/mL, in artificial urine, with daily medium exchange. The pieces were then removed, gently rinsed and analysed.

#### Crystal violet staining.

Crystal violet staining was done as described in the literature [25]. Adaptation from Kanematsu H and Barry DM. Springer Nature. 2020 (Chapter 6.1, page 114) and Colomer-Winter C et al. Bio-protocol. 2019 (page 8) with modifications [26,27]. Briefly, catheter samples were immersed in 1 mL of 0.04% crystal violet solution in PBS for 10 min, followed by three rinses in PBS with blotting towards paper in between and left to dry. For visualisation, the catheter surface was pressed against Scotch® mending tape (3M Japan, Tokyo, Japan) to transfer the biofilm. Structure of the transferred biofilm on the flat surface of the Scotch® mending tape was visualised directly or under the microscope attached to the sample glass. For quantification, biofilm was transferred on a wet (PBS) cotton swab by gently scratching the surface of the catheter piece. The head of the cotton swab was then removed and placed into 1.5 mL of 96% ethanol solution for crystal violet extraction (2h, room temperature) before spectrophotometric quantification at OD595 nm.

#### EDIC microscopy.

Qualitative assessment of biofilm development. The catheter sections were examined using an episcopic differential interference contrast (EDIC)/epifluorescence (EF) Nikon Eclipse LV100D microscope (Best Scientific, UK) using a metal halide light source (EXFO X-CITE 120 fluorescence system), long working distance metallurgical objectives (Nikon Plan Achromat) and a high-resolution camera (QImaging Retiga EXi Cooled Digital CCD monochrome camera with RGB colour filter module). Images were captured and processed using ImagePro image capture software. Experiments were repeated three times with two sections (from each experiment) used for EDIC microscopy at each time point. The entire length of the sections was examined using a x 50 objective with representative images being taken over 10–50 fields of view (including at the higher magnification of x 1000) [28].

#### Fluorescence microscopy.

For catheter surface analysis, 1 mL staining solutions of either concanavalin A (10 μg/mL, Alexa Fluor® 594 conjugate) or EbbaBiolight 680 (0.1 μg/mL) were prepared in PBS. FilmTracer™ SYPRO™ Ruby Biofilm Matrix Stain was purchased ready to use. Catheter pieces were stained for 30 min in the dark and then washed twice for 5 minutes in PBS with paper blotting in between. Fluorescent microscopy was conducted using a Trinocular Compound EPI-Fluorescence Microscope Model M837FLR (OMAX, USA) in the red and green channel mode.

#### RNA-seq analysis.

For the RNA-seq analysis, *Pseudomonas aeruginosa* PA01 strain was grown on a surface of 2-cm catheter pieces (16Fr, LubriShield^TM^ vs uncoated silicone catheter) in 1 mL of AUM in microcentrifuge tubes for 7 days at 37°C with slow shaking and daily changing of the medium. Catheter pieces were gently removed and thoroughly rinsed 5 times in PBS with liquid carefully removed in between. After that, they were frozen in pre-chilled 2-cm tubes and stored at −80°C or in dry ice during transportation prior to the RNA extraction procedures. RNA extraction, library construction, prokaryotic transcriptome sequencing was performed by BGI Genomics (Hong Kong, China). Total RNA was isolated. The library was constructed in the following steps: a) rRNA depletion, b) RNA fragmentation, c) first cDNA strand synthesis, d) second cDNA strand synthesis, e) end repair and adaptor ligation, f) PCR amplification, g) library purification and qualification. After that sequencing was performed using DNBSEQ sequencing platform. Sequencing reads were filtered, mapped to the published Pseudomonas aeruginosa PA01 genome software [29] using HISAT2 [30] and StringTie software for assembly [31]. Bowtie2 was used to align clean reads to the reference sequence [32], and RSEM was used to calculate the expression of genes and transcripts [33]. For the differential analysis between groups, DEGseq method was used [34] and between samples Poisson distribution method was used [35]. Hierarchical clustering analysis of DEGs, gene ontology analysis of DEGs and pathway analysis of DEGs was performed. For mRNA, annotation following databases were used: NR, NT, GO, KOG, KEGG, Uniprot, COG, PRG, String, TF. For lncRNA, the following annotated databases were used: miRBase, Rfam. Hierarchical clustering analysis of DEGs, gene ontology analysis of DEGs and pathway analysis of DEGs was performed.

#### Antibiotic sensitivity assays.

For the antibiotic sensitivity assay 7-day biofilm cultures of bacteria were prepared on LubriShield^TM^ coated vs. uncoated 2-cm catheter pieces as it is described above. The pieces were thoroughly washed from loosely adhered bacteria (3x, 1 min washes in 1 mL of PBS with paper blotting in between and placed into 1 mL of fresh AUM to recover for 1h at 37°C with shaking (90 rpm). a) For the colistin sensitivity assay: three pieces of each kind were then treated with colistin (16 μg/mL in AUM, 37°C, 2h), while another triplicate was used as the untreated reference. After the treatment, Cell Disruptor (AOSENR, China) was used to remove adhered bacteria and disrupt biofilm (30 seconds sonication/ 10 minutes cooling/ 30 seconds sonication). 10x serial dilutions were made and 100 µL of each of them were placed on Luria agar plates and spread with sterilised glass beads. Next day, colonies were counted and CFU/mL in the original suspension calculated. b) For the vancomycin sensitivity assay: three pieces of each kind were treated with vancomycin (25 μg/mL in AUM, 37°C, 2h), while another triplicate was used as the untreated reference. 50 µL of the WST working solution was added to each of the tubes and incubated for the additional 1h at 37°C. 400 µL sample was then spun down to remove debris and OD450 nm measured with spectrophotometer.

### Statistical analysis

Biofilm formation measured by crystal violet comparing LubriShield^TM^ catheter surface with control silicone catheter surface was analysed by Student’s unpaired, two-tailed t-test. Viability data by CFU/mL were log-transformed to achieve a normal distribution. The log reduction of each experiment was determined by subtracting the log CFUs before treatment from the log CFUs after treatment. Data are plotted as the means with staple bars showing the standard error of the mean (SEM). The probability value less than 0.05 was considered significant. The calculations were performed using Microsoft Office Excel 2023.

## Results

### The LubriShield^TM^ coated surface prevented multiple uropathogenic strains from forming biofilm

The ability of different strains of the most common uropathogens to form biofilm in artificial urine media (AUM) on urinary catheters was tested under static condition at 37° after 5 and up to 14 days. Coated and uncoated silicone urinary catheters were aseptically cut into 2-cm lengths and individual pieces were inoculated with 10^9^ CFU/mL of the different uropathogens in AUM. Culture media was replaced every day to provide fresh nutrients and to discard unattached bacteria. After 7 days, all uropathogens had formed thick biofilms on the control silicone catheter surfaces. Representative fluorescence microscopy images show that the LubriShield^TM^ coated catheters prevented adhering *K. pneumoniae*, *E. faecium*, and *C. albicans* producing detectable amounts of polysaccharides on the surface determined by staining with Alexa 549-coupled fluorescent concanavalin A (Fig 2A). Further staining with a non-specific protein fluorochrome, SYPRO Ruby, revealed that in contrast to the uncoated silicone catheter surface, no protein components of the EPS were found on the LubriShield^TM^ coated surface regardless of which uropathogenic microbe that was incubated in the AUM (Fig 2B).

Concentrated inoculations with single and mixed strains of *E. coli, K. pneumoniae, S. aureus, P. mirabilis and E. faecalis* (10^9^ CFU/mL) were cultured on catheter pieces for 14 days in AUM. Quantification of the adherent biomass, including cells and EPS, was assessed by the crystal violet staining method. The absorbance of the extracted staining showed that still after 14 days, there was a significantly reduced biofilm formation for all tested uropathogens on the LubriShield^TM^ catheter surface compared to the silicone surface (Fig 2C) (ranging from *P = 0.009* to *P < 0.001*). Scotch tape captures of the crystal violet staining showed how the LubriShield^TM^ catheter surface was clean even after inoculation with common mixed-population biofilm species such as *E. coli* and *E. faecalis* or *P. mirabilis* and *E. faecalis* (Fig 2C).

Uncoated and coated catheters were incubated with exponentially grown liquid culture of the uropathogenic *P. mirabilis* in AUM. There were no significant differences in pH values between the cultures containing the uncoated silicone and LubriShield^TM^ coated catheters. In both cases considerable increase to pH 9.0-9.5 was seen within 24 hours, and then maintained through the duration of the experiment. Catheter samples were also examined using EDIC/EF microscopy. EDIC imaging uses long working distance, non-contact, high magnification objectives to allow non-destructive imaging of biofilm structures and their interactions with surface materials [28]. The appearance of crystals, particularly struvite’s, were observed in all samples as expected with the observed increase in pH. The internal and external catheter surfaces of the uncoated control sample showed a thick biofilm covering big areas in a sheath-like structure (Fig 2D). In contrast, the LubriShield^TM^ coated catheters showed a patchier, mosaic-like pattern, indicating microcolonies of bacteria. Assessing the biofilm structure using the EbbaBiolight stain, which labels amyloid proteins and negatively charged polysaccharides with repeating units in the EPS, showed only signal on the uncoated silicone surface as well as on crystalline formations that cause adsorption of the stain (Fig 2D).

### The LubriShield^TM^ coating did not exert any microbial killing effect

Quantification of the attached bacteria by culture techniques did not show any significant differences between the uncoated and coated samples as seen in Fig 2E. In all cases, the colony-forming ability was maintained during the seven-day culture. This confirmed that there was no bactericidal or bacteriostatic effect of the LubriShield^TM^ coating (Fig 2E).

To analyse if the LubriShield^TM^ coating had a permanent biofilm-preventive effect on bacteria, uncoated and coated catheter pieces were inoculated with 10^9^ CFU/mL of either *K. pneumoniae* or *S. epidermidis* for 7 days in AUM with daily medium replacement. The catheter-adhering bacteria were sonicated off, counted and transferred to new tubes with uncoated catheter pieces and cultured again for seven days in AUM. Biofilm formation was assessed by staining all catheter surfaces with crystal violet and the extracted staining was then read in a spectrophotometer. The results showed that although biofilm formation by adhering bacteria was significantly inhibited on the LubriShield^TM^ coated catheter surfaces, the same bacteria were able to form biofilm on uncoated catheters equal to the biofilm-forming bacteria coming from the uncoated surfaces (Fig 2F).

### A strong anti-biofilm effect occurred even under dynamic conditions

**Fig 3:**
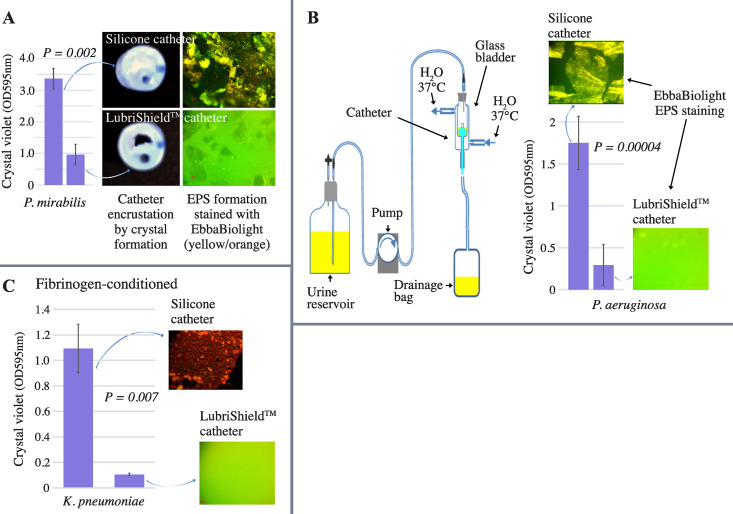
Biofilm assessment of the LubriShield^TM^ catheter in dynamic flow models. (A) At the end of the incubation, the control silicone catheter lumen was completely blocked with crystal precipitation in contrast to the LubriShieldTM coated catheter lumen (left panel). Crystal formation (middle panels) and fluorescent labelling of biofilm (right panels, magnification X 40) on LubriShield^TM^ surface after *P. mirabilis* cultured in AUM for 3 days in a drip flow reactor. Biofilm biomass was quantified using crystal violet staining (left panel). (B) Schematic illustration of the in vitro glass bladder model system (left panel). Water was circulated through the outer chamber of a double walled vessel to maintain the glass bladder at 37°C. Catheters were inserted into the inner chamber and retention balloons inflated to hold the catheter in place. Catheters were connected to standard drainage bags below the level of the vessel to form a complete sterile closed drainage system. AUM was supplied to the glass bladder from the top at a constant flow rate controlled by a pump [36]. Right panel shows crystal violet counts and fluorescent staining of the catheter sections from a glass bladder experiment after 5 days of *P. aeruginosa* culture in AUM. (C) Catheters with or without LubriShield™ coating were preconditioned with fibrinogen and incubated for 7 days with *K. pneumoniae* in artificial urine. Biofilm biomass was quantified using crystal violet staining. Bars represent the mean ± SEM (n = 3) and *P* value from Student’s t-test. Fluorescent microscopy images (40X magnification, stained with a combination of EbbaBiolight 680 and ConA-AF594) illustrate biofilm formation differences between uncoated silicone and LubriShield™-coated catheters.

We challenged the ability of uropathogens to form catheter-associated biofilms during dynamic conditions in a flow model. Uncoated and coated catheters were inoculated with urease-positive *P. mirabilis* (10^9^ CFU/mL) and incubated at 37°C under a pulsing flow of AUM for 3 days. At the end of the test, the control silicone catheter lumen was completely blocked with crystal precipitation in contrast to the LubriShield^TM^ coated catheter lumen, where less crystals had formed (Fig 3A). Labelling of EPS on the same catheter surfaces with EbbaBiolight 680, showed that the biofilm formation covered almost all of the control silicone catheter surface contrary to the LubriShield^TM^ coated catheter surface. Significantly less biofilm could be spotted (71% reduction, *P = 0.002*) predominantly associated with biomass present on the surface of crystals (Fig 3A).

To further consider the hydrodynamic influence of the urine flow on the development of catheter-associated biofilms, we compared the whole length of the LubriShield^TM^ coated silicone catheters with uncoated catheters in an *in vitro* glass bladder model system (Fig 3B, left panel). Six bladders with three LubriShield^TM^ coated catheters, and three uncoated catheters were set up to study the biofilm formation of *P. aeruginosa*. A peristaltic pump provided AUM flow through the catheter at a rate of 1.5 mL/min and the temperature was held at 37°C. After five days, the models were stopped, and the catheters were analysed.

Formation of biofilm on the luminal surfaces of the catheters was assessed by labelling EPS with EbbaBiolight 680 for microscopic determination and crystal violet staining for quantitative measurements. Results calculated from the mean crystal violet absorbance of three catheter replicates showed an 83% significant reduction (*P = 0.00004*) in biomass on the LubriShield^TM^ coated catheter compared to the uncoated catheter (Fig 3B, right panel). Fluorescence microscopy imaging of the EPS formation confirmed the anti-biofilm effect of the LubriShield^TM^ coating (Fig 3B, right panel).

### Fibrinogen preconditioning does not impair LubriShield™‘s antibiofilm performance

To assess whether host-derived proteins could influence LubriShield™‘s antibiofilm efficacy, coated and uncoated silicone catheters were preconditioned for one hour with plasma-level concentrations of fibrinogen (1 mg/mL) prior to incubation with *K. pneumoniae* in artificial urine for 7 days under static conditions. Despite fibrinogen preconditioning, LubriShield™ significantly reduced biofilm formation compared to uncoated silicone catheters (Fig 3C, *P = 0.007)*. Fluorescent microscopy confirmed extensive EPS release on fibrinogen-conditioned silicone surfaces, whereas LubriShield™ surfaces demonstrated minimal amounts of EPS. These findings demonstrate that LubriShield™ effectively inhibits biofilm formation even in the presence of host proteins known to facilitate microbial attachment.

### Reduced biofilm-associated gene expression on the LubriShield ^TM^ coated catheter

**Fig 4:**
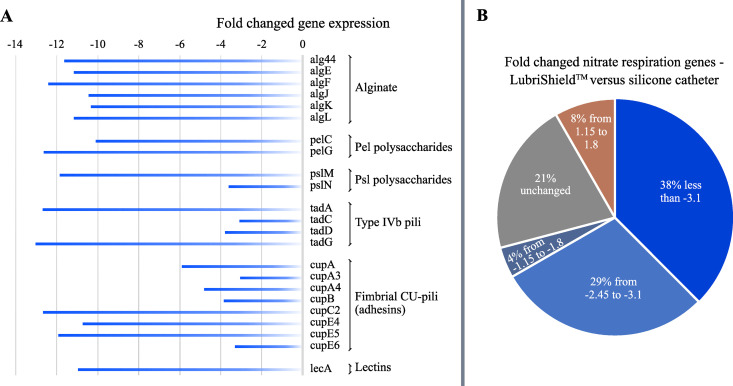
Transcriptome of surface-adhering bacteria after 7-day culture in AUM. (A) Gene expression analysis of LubriShield^TM^ adhering *P. aeruginosa* showed significant down-regulation of biofilm-associated EPS operons compared to uncoated silicone as analysed by RNA-seq and visualised via volcano plots (S2 Fig). (B) Pie chart showing fold-change in anaerobic nitrate respiration genes expression in LubriShield^TM^ adhering *P. aeruginosa* versus uncoated silicone catheters. Shades of blue indicate reduced expression, orange indicates increased expression, and grey represents unchanged genes. Specific genes and fold-changes are detailed in S4 Table.

Cells in biofilm mode manifest different gene expression pattern compared to the planktonic ones. *Pseudomonas aeruginosa* PAO1, a well-studied laboratory strain with a sequenced genome and often used as a model for biofilm-related gene analysis [37,38], was used to compare gene expression on mRNA level in bacteria growing for seven days on the LubriShield^TM^ coated surface in comparison to uncoated silicone catheter using RNA-seq analysis. Significant difference (using volcano plot) in the mRNA transcript was observed, with more than half of all the genes changing expression between the two groups (S2 Fig). We conducted analysis of the mRNA expression levels of key EPS-related operons. As Fig 4A shows, the expression levels of several key genes in those operons were significantly higher on the un-coated surface compared to LubriShield^TM^. In particular the genes responsible for the production of alginate (*alg44* (PA3542), *algE* (PA3544), *algF* (PA3550), *algK* (PA3550), *algL* (PA3547); pel polysaccharide (*pelC* (PA3062), *pelG* (PA3058); psl polysaccharide (*pslM* (PA2243), *pslN* (PA2244); tight adherence proteins constituting type IVb pili (*tadA* (PA4302), *tadC* (PA4300), *tadD* (PA4299), *tadG* (PA4297)); fimbrial CU-pili adhesins (*cupA1* (PA2128)*, cupA3* (PA2130)*, cupA4* (PA2131)*, cupB1* (PA4086)*, cupC2* (PA0993)*, cupE4* (PA4651)*, cupE5* (PA4652)*, cupE6* (PA4653); and lectins, *lecA* (PA2570)) were found to be upregulated.

The most striking difference was observed for the genes involved in the nitrate respiration, common among bacteria residing at the bottom of biofilms. As can be seen in Fig 4b, 67% of the nitrate respiration genes had significantly higher mRNA expression levels on the uncoated silicone catheter surface compared to the LubriShield^TM^ coated one. Most notably, 38% of genes had dramatically reduced expression (< −3.1 fold), and 29% moderately reduced (−2.45 to −3.1 fold). In addition, genes involved in arginine fermentation, which is another sign of anaerobic lifestyle in *Pseudomonas,* as well as other hypoxia-related genes [39] had significantly higher levels on the uncoated silicone surface (S4 Table). This finding indicated that a significant proportion of the cells were in an anaerobic environment, a characteristic of complex biofilm structure, while no such structure was present on LubriShield^TM^ coated catheters.

### Increased antibiotic response of bacteria present on LubriShield^TM^ coated catheters

To assess if the biofilm-preventive effect of the LubriShield^TM^ coating also included reducing antibiotic recalcitrance, two common biofilm-forming uropathogenic strains, one Gram-negative and one Gram-positive, were tested in 7-day long cultures in artificial urine on LubriShield^TM^ coated catheters compared to uncoated control catheters. Two different strategies were used: i) to treat with a bactericidal antibiotic (colistin) and then remove bacteria for subsequent viable count determination, ii) to directly assess metabolic status of the cells after a bacteriostatic antibiotic treatment (vancomycin), without removing cells from the catheter surface.

**Fig 5:**
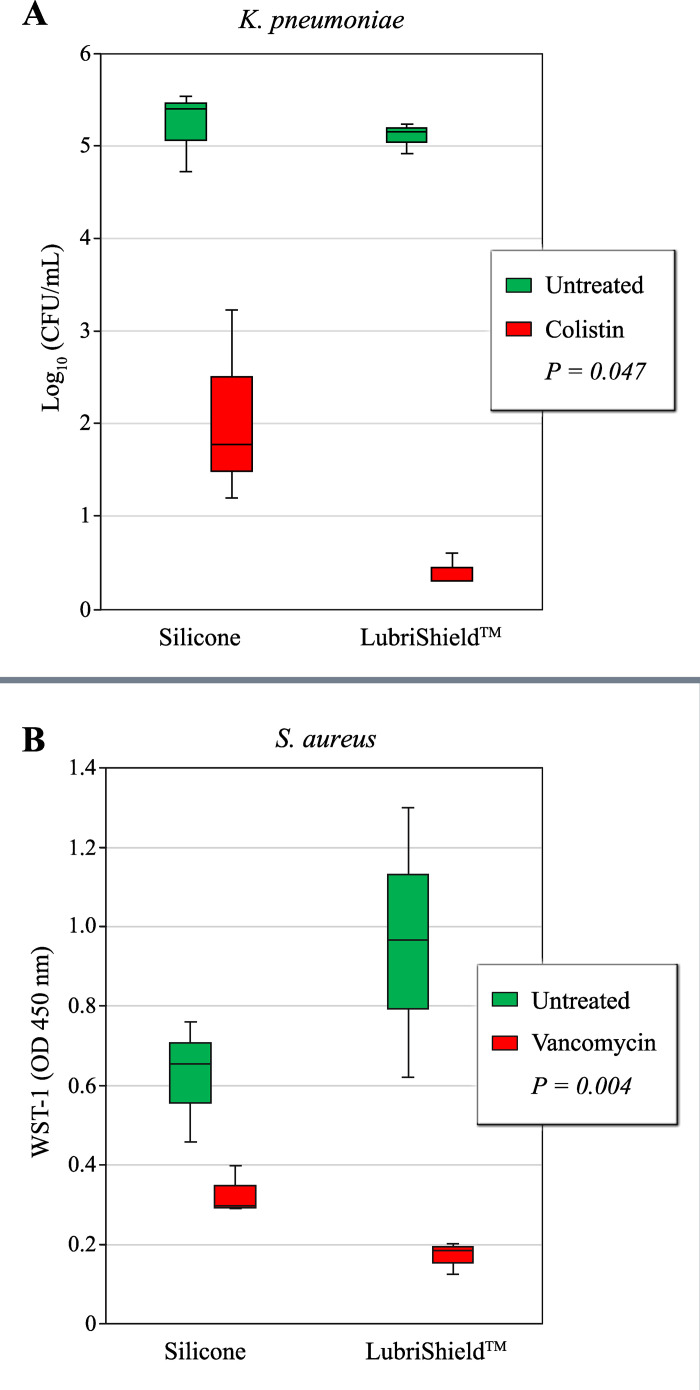
Treatment response to antibiotics after 7 days biofilm culture on catheters. (A) Quantification of catheter-adhering *K. pneumoniae* by log_10_ (CFU/mL) count on uncoated Control versus LubriShield^TM^ coated catheters treated with colistin (red) or no treatment (green) (n = 3). (B) Evaluation of metabolic activity by measuring WST-1 of catheter-adhering *S. aureus* on uncoated control- versus LubriShield^TM^ coated catheters treated (green) with vancomycin or no treatment (red). P-values from Student’s t test comparing treatment-induced effect (n = 3) are depicted.

In the first case, after seven days with fresh daily media change at biofilm formation conditions, adhering *K. pneumoniae* were challenged with colistin (polymyxin E, 16 µg/mL) or left untreated for 2 hours and then assessed for viable counts. Results depicted in the boxplot Fig 5A show a significantly improved response to treatment of bacteria present on the LubriShield^TM^ coated catheter compared to the uncoated catheter (Fig 5A, reduction from log_10_(3.2) to log_10_(4.7), *P = 0.047)*.

To test if the LubriShield^TM^ coating also resulted in an increase in the antibiotic responsiveness in Gram-positive bacteria, coated and uncoated control catheters were inoculated with *S. aureus*. After 7 days of culture in AUM, loosely bound bacteria were rinsed off and catheter pieces were treated for 2 hours with vancomycin (25 µg/mL) with identical untreated pieces serving as a reference. Reduction of the nontoxic tetrazolium salt, WST-1, which correlates to the cellular respiration rate, was then analysed with a spectrophotometer [40]. Results depicted in Fig 5B show 33% lower WST-1 signal from the adhering bacteria on uncoated compared to LubriShield^TM^ coated catheter pieces (green boxes). This phenomenon is often associated with biofilm-living bacteria known to reduce their metabolism. After antibiotic treatment, 82% of the metabolic activity of the LubriShield^TM^ coated catheter-adhering bacteria was abolished, while a significantly less reduction (46%) was seen in bacteria present on the uncoated control-catheters (*P = 0.004*).

Collectively, these findings from morphological analyses, RNA-seq data, and antibiotic sensitivity assays strongly indicate that bacteria adhering to the LubriShield™ coating do not sense the need to switch into biofilm mode. Consequently, they remain functionally similar to planktonic bacteria, explaining their increased susceptibility to clinically relevant antibiotics.

### The LubriShield^TM^ coating generated a permanent, non-release and non-toxic surface with remarkable lubrication and wettability

**Fig 6:**
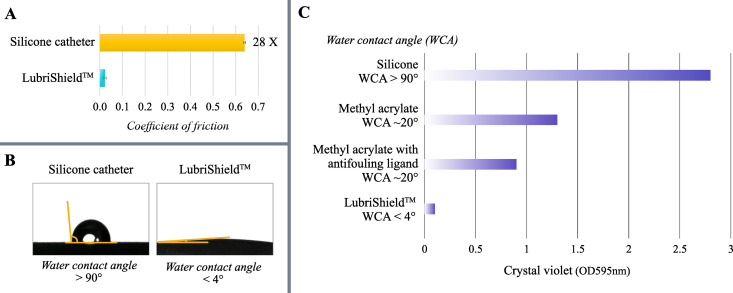
Physical characteristics of the coating. (A) Sliding friction analysis comparing wetted surfaces of uncoated silicone and LubriShield^TM^ coated catheters, presented as the coefficient of friction. (B) Water contact angle (WCA) measurements of catheter surfaces demonstrating hydrophilicity, comparing LubriShield^TM^ coated (WCA < 4°) and uncoated silicone (WCA > 90°) catheter surfaces. (C) Biofilm quantification (crystal violet assay) after 7-day culture of *K. pneumoniae* in artificial urine, comparing uncoated silicone catheters (WCA > 90°), modified coatings with hydrophobic methyl acrylate (WCA ~ 20°), methyl acrylate with antifouling ligand (WCA ~ 20°), and LubriShield^TM^ coating (WCA < 4°).

Immediately upon wetting in water or artificial urine medium, the LubriShield^TM^ coated Foley catheter exhibited significantly reduced friction. The coefficient of friction (CoF) for LubriShield^TM^ was measured to be 28 times lower (CoF = 0.02) compared to uncoated catheters (CoF = 0.64), as illustrated in Fig 6A, *P < 1 x 10*^*−7*^).

The wettability of LubriShield^TM^ was further characterised using the sessile drop technique, where the water contact angle (WCA) indicates the degree of surface hydrophobicity. Surfaces with WCA greater than 90° are considered hydrophobic, whereas those below 90° are hydrophilic [41]. A superhydrophilic surface, defined by a WCA of less than 10°, rapidly spreads water across its surface. LubriShield^TM^ demonstrated a superhydrophilic property with a WCA of less than 4°, compared to the hydrophobic uncoated silicone catheter surface (WCA > 90°) (Fig 6B).

To illustrate the critical importance of LubriShield^TM^’s superhydrophilic nature and antifouling ligand in preventing biofilm formation, we modified the coating by incorporating the hydrophobic monomer methyl acrylate, both with and without the antifouling ligand. The WCA for these modified coatings increased to approximately 20°. Fig 6C presents biofilm formation quantification via crystal violet staining after a 7-day incubation with *K. pneumoniae* in artificial urine. The uncoated silicone catheters (WCA > 90°) demonstrated significant biofilm formation. Methyl acrylate modification alone (WCA ~ 20°) reduced biofilm formation by 54% compared to silicone. The addition of the antifouling ligand further reduced biofilm formation by an additional 30%. Most notably, LubriShield^TM^ without modifications (WCA < 4°) showed almost no biofilm formation.

These findings highlight the LubriShield^TM^ coating’s unique combination of extreme wettability and effective antifouling properties, providing robust, long-lasting protection against biofilm formation on catheter surfaces.

### LubriShield^TM^ is safe, does not release any toxic compounds and has no antimicrobial or cytotoxic effect

To assess the safety and biocompatibility of the LubriShield^TM^ coated catheter, we performed various *in vitro* tests. The chemical characterisation of the coated catheter revealed that no chemical-substance release from the coating could be detected (ISO 10993–18:2020 and ISO 10993–12:2021). In addition, extended duration of bacterial cultures on the LubriShield^TM^ coated catheter, showed no antimicrobial or bactericidal effect (Fig 2E) and thus did not induce any host reactions against toxic bacterial debris. Furthermore, cytotoxicity tests of the LubriShield^TM^ coating were performed on endothelial cells according to ISO standard 10993−5 and showed negligible cytotoxicity against human bladder and uroepithelial cells (Table 1). The skin irritation assessment of the LubriShield^TM^ coating was also negative according to the ISO standard 10993−5 (Table 1). These results confirmed that the LubriShield^TM^ coated Foley catheter was without toxicity, and compatible with biological systems.

**Table 1:** Cytotoxicity testing according to ISO 10993−5.

*Cytotoxicity test of extract*	No cytotoxic potential
***EpiDerm skin irritation test***	No skin irritation potential
***Cytotoxic test of direct contact to L929 mouse cells***	No cytotoxic potential
***Cytotoxic test of direct contact to human uroepithelial/bladder cells***	No cytotoxic potential

## Discussion

Catheter-associated urinary tract infections (CAUTIs) continue to be among the most prevalent healthcare-associated infections in both hospital and community care settings [42]. Even with the most scrupulous nursing care and rigorous application of sterile closed drainage systems, bacteriuria is almost inevitable in patients undergoing long-term catheterization (> 28 days), and this group of patients are particularly vulnerable to resulting complications [43]. Currently, coatings on urinary indwelling catheters that release antimicrobial compounds (silver alloy or antimicrobials) have only been reported to yield positive results for short-term use.

Adherence of bacteria and fungi is a key step in infection; once adherent, the microorganisms can colonise and form biofilm often leading to CAUTI or blockage of the catheter lumen. It is therefore an urgent need to develop coatings on indwelling catheters that can prevent or reduce the formation of biofilms including crystalline biofilms. The common property of biofilms of different species is the presence of a matrix surrounding the community, shaping and protecting it, as well as, immobilising its members. Microbial adhesion leading to biofilm formation is a complex process controlled by the interplay between physicochemical, mechanical, topographical surface properties, characteristics of the microorganisms, and environmental conditions. Most biofilm studies overlook the influence of surface parameters and surrounding hydrodynamic conditions on bacterial sensing and binding behaviour to a substrate. When bacteria adhere to a surface, the adhesion forces exerted by the substratum can induce nanoscopic deformation of the bacterial cell wall, leading to changes in the intracellular cytoplasmic pressure [19,44]. Membrane-located sensor molecules subsequently react, which translates into a biological response with the synthesis and secretion of EPS [19,44]. Superhydrophilic surfaces create a dense layer of water molecules, which weakens the interaction between cell surfaces and substratum material, thereby reducing cell adhesion [45,46]. Studies have demonstrated that both Gram-positive and Gram-negative bacteria produce less EPS and are more susceptible to antibiotics on hydrophilic surfaces compared to hydrophobic surfaces with stronger adhesion properties [47–49]. Similar behaviour has been observed with *C. albicans* biofilm formation on surfaces with high wettability [50,51]. In addition to surface properties, hydrodynamic conditions, such as urine flow, play a critical role in biofilm development. The shear stress exerted by urine flow stimulates uropathogens to enhance their adhesion by producing thicker, more resistant biofilms as a countermeasure [52]. Catheter encrustation caused by urease-producing organisms is also aggravated under these conditions and represents a serious complication for patients with indwelling catheters [53]. As a result, indwelling urinary catheters provide an ideal environment for biofilm formation, significantly increasing the risk of recalcitrant clinical infections. In this study, we demonstrate the effectiveness of the LubriShield™ coating in preventing biofilm formation under both static and dynamic flow conditions.

Biofilm-related infections are notoriously resistant to a broad range of antibiotics and biocides, posing a significant clinical challenge due to persistent symptoms despite prolonged antimicrobial treatments. This recalcitrance is attributed to biofilm-specific features, including i) EPS-induced tolerance, ii) gene-regulated resistance mechanisms, and iii) dormant persister cells [54–56]. In this study, we evaluated the susceptibility of catheter surface-associated bacteria after 7 days of culture in AUM to two common antibiotics used for treating complicated urinary tract infections. Our results demonstrated that biofilm-forming *K. pneumoniae* on LubriShield^TM^ coated surfaces were significantly more responsive to colistin, an antibiotic commonly used for multidrug-resistant Gram-negative infections, compared to uncoated silicone catheter surfaces. Similar results were shown for vancomycin, the therapy of choice for difficult-to-treat staphylococcal infections, which was significantly better at reducing the metabolic state in *S. aureus* after 7 days of culture on LubriShield^TM^ than on silicone catheter surface. The observed increase in antibiotic sensitivity among bacteria adhering to LubriShield™ coated catheters is particularly important. Typically, bacterial biofilms exhibit markedly increased recalcitrance to antibiotics due to the protective extracellular polymeric substance (EPS) barrier, metabolic dormancy, and altered gene expression. Our RNA sequencing results, supported by microscopic observations demonstrating absence of EPS, reveal that bacteria adhering to the LubriShield™ surface do not initiate biofilm-related genetic pathways. Consequently, they maintain a planktonic-like state even while physically attached. This finding has critical implications for clinical management, suggesting that catheter-associated bacterial colonisation in the presence of LubriShield™ could remain controllable with antibiotic treatment without necessitating catheter removal.

With growing evidence that biofilm formation plays a major role in medical device-related infections, updating detection standards to reflect advances in our understanding of biofilm composition is essential for accurate assessment and effective treatment. Existing standards, such as live/dead fluorescent staining, bioluminescent assays detecting ATP consumption, and culture-based analysis of colony-forming units (CFUs), primarily target metabolically active bacteria. These methods are therefore better suited for evaluating bactericidal antimicrobials. However, these assays often produce false-negative results when analysing bacterial biofilms due to the presence of dormant persister cells with exceptionally low growth rates [57]. To reliably differentiate planktonic bacteria from biofilm-forming bacteria, assays must target the extracellular polymeric substance (EPS) components, as planktonic bacteria do not produce EPS. The most widely used method is the colorimetric crystal violet (CV) assay, which binds to negatively charged components of the EPS – such as polysaccharides, proteins, extracellular DNA (eDNA), and lipids – as well as to live and dead bacteria. The CV assay’s simplicity, broad detection spectrum, and quantifiability make it ideal for *in vitro* biomass assessment. However, evaluation of medical devices explanted from patients can be confounded by host-induced protein responses that interfere with results [58]. Microscopic imaging techniques, such as episcopic differential interference contrast (EDIC) microscopy, provide an excellent way to examine the morphology of colonised surfaces *ex situ*, revealing the spread and structure of biomass sheaths or patches [28]. Fluorescent tagging of specific EPS components further enhances biofilm detection, enabling precise confirmation of biofilm presence and composition. Finally, since biofilm formation and its emergent properties are regulated by genes responsive to surface sensing, stress, and quorum sensing signals, RNA-seq analysis is a powerful tool. It provides a detailed snapshot of the gene expression profile of the colonising microbial population, offering critical insights into the biofilm-forming state [7,59].

The LubriShield^TM^ coating is designed to create a biocompatible, non-releasing, superhydrophilic, and permanent surface without exhibiting any microbial killing effects. Microbial incubation in artificial urine demonstrated significant inhibition of biofilm formation by the 8 most prevalent uropathogenic microbes under both static and dynamic conditions. The coating’s near-zero water contact angle was directly associated with its unique antifouling properties. Our findings suggest that interfering with bacterial surface-sensing mechanisms is critical for preventing the transition to a biofilm-forming state and the activation of biofilm-specific RNA expression profiles. Notably, no antimicrobial or bactericidal effects were observed in cultures of extended duration, and chemical characterisation confirmed the absence of any substance release from the coating. In addition to its antifouling properties, the LubriShield™ coating exhibited a 28-fold reduction in surface friction compared to uncoated silicone catheters, further highlighting its potential to improve clinical outcomes by reducing mechanical irritation and biofilm formation.

Overall, these experimental findings suggest that the novel LubriShield^TM^ coating has the potential to significantly improve clinical outcomes by reducing patient discomfort, the risk of urethral stenosis, encrustation, and CAUTIs. Importantly, fibrinogen preconditioning experiments showed that LubriShield™ maintained excellent antibiofilm activity despite the presence of host protein conditioning, a key step in catheter-associated biofilm formation.

Furthermore, the results indicate that catheter-related bacterial infections could potentially be treated with antibiotics without the need to remove the catheter. While these preclinical findings are promising, clinical studies are necessary to confirm their applicability in vivo. Ongoing Phase I and Phase II trials aim to evaluate the safety, efficacy, and clinical utility of LubriShield™ coated catheters in patient populations.

## Supporting information

S1 Fig Volcano plot representing all expressed transcripts. For every transcript, the fold change of LubriShield^TM^ versus silicone catheter-associated *P. aeruginosa* was plotted against the -log P value. Statistically significant differentially expressed genes, with a fold change ≥1.5 or ≤ −1.5, are depicted as red, insignificant as black dots.
(PDF)

S2 Fig Crystal violet staining of an uncoated and a coated Foley catheter. The uniformity of the grafted surfaces of the silicone catheters was analysed by staining in an aqueous solution containing methanol and Crystal violet (4%).
(PDF)

S1 Table Summary of chemical characterisation of LubriShield^TM^ catheter. (PDF)

S2 Table Chemicals used for artificial urine medium preparation. (PDF)

S3 Table Bacterial and fungal strains used in the study. (PDF)

S4 Table Fold change in gene expression related to nitrate respiration, arginine fermentation and other hypoxia-related genes in LubriShield^TM^ versus silicone catheter-associated *P. aeruginosa.* (PDF)
