# Supplementary material for: LubriShieldTM—A permanent urinary catheter coating that prevents uropathogen biofilm formation in vitro independent of host protein conditioning
Source: PLoS One. 2025 Jul 10;20(7):e0328167. doi: 10.1371/journal.pone.0328167 (PMC12244716; doi:10.1371/journal.pone.0328167)
Supplement: S1 Fig — For every transcript, the fold change of LubriShieldTM versus silicone catheter-associated P. aeruginosa was plotted against the -log P value. Statistically significant differentially expressed genes, with a fold change ≥1.5 or ≤ −1.5, are depicted as red, insignificant as black dots. (PDF) [file pone.0328167.s001.pdf]

Standard
silicone
catheter

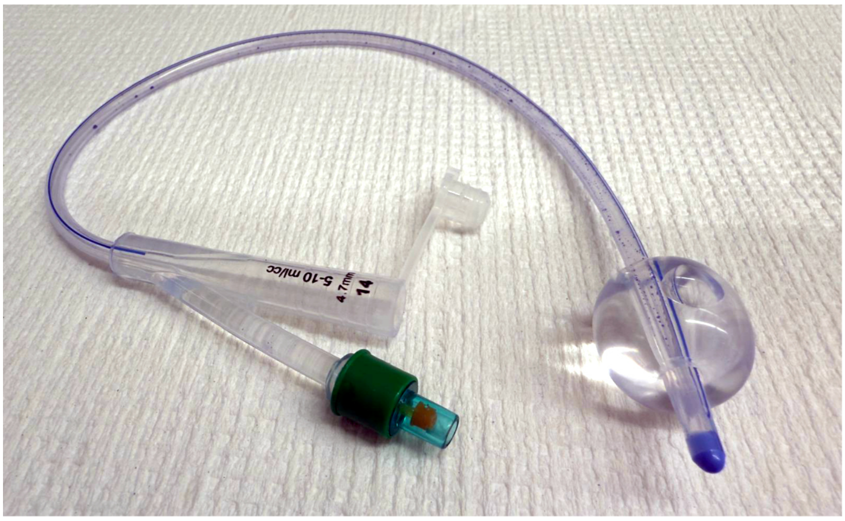

LubriShield™
catheter

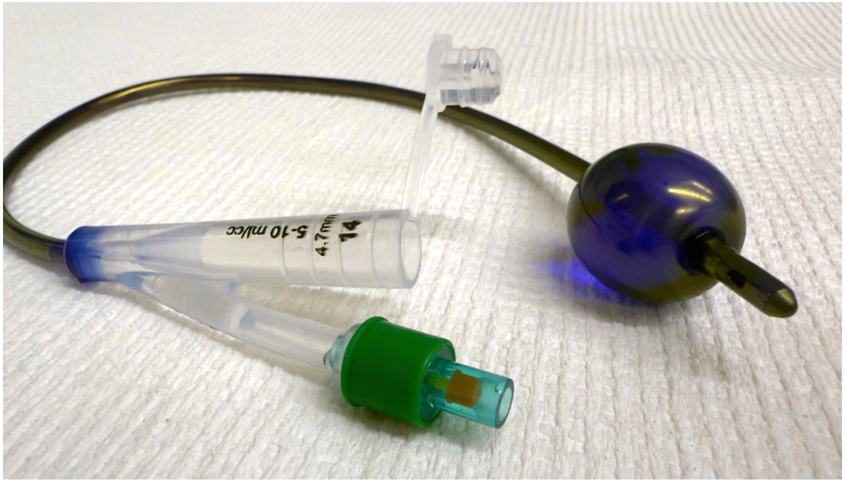

S1 Fig. Crystal violet staining of an uncoated and a coated Foley catheter. The uniformity of the grafted surfaces of the silicone catheters was analysed by staining in an aqueous solution containing methanol and Crystal violet (4%).
