# Supplementary material for: LubriShieldTM—A permanent urinary catheter coating that prevents uropathogen biofilm formation in vitro independent of host protein conditioning
Source: PLoS One. 2025 Jul 10;20(7):e0328167. doi: 10.1371/journal.pone.0328167 (PMC12244716; doi:10.1371/journal.pone.0328167)
Supplement: S2 Fig — The uniformity of the grafted surfaces of the silicone catheters was analysed by staining in an aqueous solution containing methanol and Crystal violet (4%). (PDF) [file pone.0328167.s002.pdf]

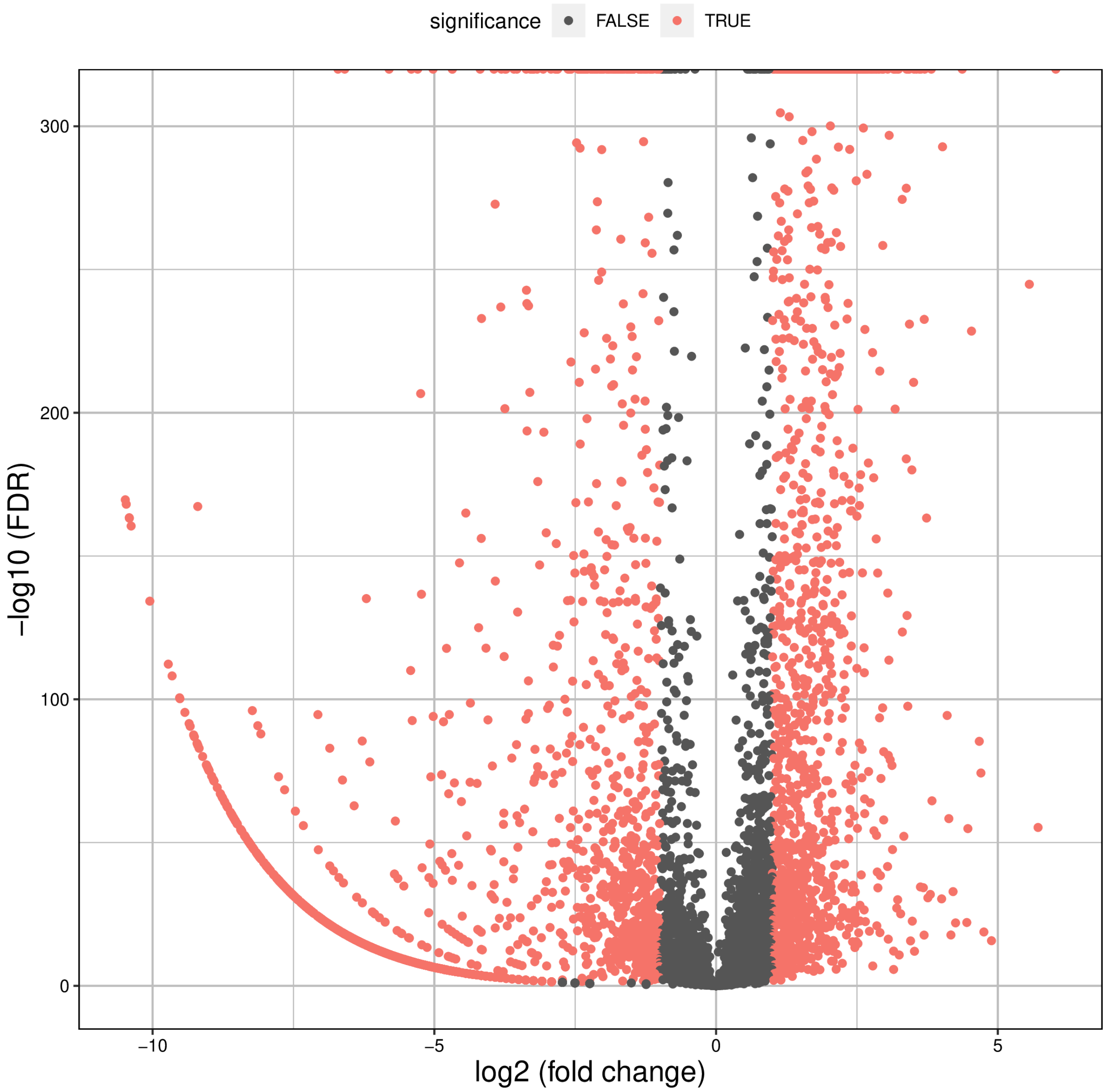


***S2 Fig. Volcano plot representing all expressed transcripts.*** For every transcript, the fold change of LubriShield$^{TM}$ versus silicone catheter-associated *P. aeruginosa* was plotted against the -log P value. Statistically significant differentially expressed genes, with a fold change ≥1.5 or ≤ -1.5, are depicted as red, insignificant as black dots.
