# Supplementary material for: LubriShieldTM—A permanent urinary catheter coating that prevents uropathogen biofilm formation in vitro independent of host protein conditioning
Source: PLoS One. 2025 Jul 10;20(7):e0328167. doi: 10.1371/journal.pone.0328167 (PMC12244716; doi:10.1371/journal.pone.0328167)
Supplement: S1 Table — (PDF) [file pone.0328167.s003.pdf]

**S1 Table. Summary of chemical characterisation of the LubriShield™ catheter**

<table>
<tr><th>Type of Extractable</th><th>Test Parameter</th><th>Extraction Conditions</th><th>Analytical Methods</th><th>Analytical Evaluation Threshold (AET, µg/ device)</th><th>Result</th></tr>
<tr><td rowspan="3">Organic</td><td>Volatile Organic Compounds (VOCs)</td><td rowspan="4">Exhaustive extraction (3 $cm^2$/ml, at 50°C, 72h) with water, isopropanol, n-hexane (polar, semi-polar, nonpolar solvents)</td><td>GC-MS Headspace</td><td>10</td><td rowspan="4">No substances of coating components detected above AET</td></tr>
<tr><td>Semi-Volatile Organic Compounds (SVOCs)</td><td>GC-MS/MS</td><td>10</td></tr>
<tr><td>Non-Volatile Organic Compounds (NVOCs)</td><td>LC-MS/MS</td><td>2</td></tr>
<tr><td>Inorganic</td><td>Elemental impurities</td><td>ICP-MS</td><td>Determined by TTC (1.5 µg/day)</td></tr>
</table>

*All tests were performed according to ISO 10993-18 AMD-1:2022 and ISO/TS*
