# Supplementary material for: LubriShieldTM—A permanent urinary catheter coating that prevents uropathogen biofilm formation in vitro independent of host protein conditioning
Source: PLoS One. 2025 Jul 10;20(7):e0328167. doi: 10.1371/journal.pone.0328167 (PMC12244716; doi:10.1371/journal.pone.0328167)
Supplement: S2 Table — (PDF) [file pone.0328167.s004.pdf]

S2 Table. Chemicals used for artificial urine medium preparation

Chemical	CAS	Producer
Urea	57-13-6	Carlo Erba, Italy
Magnesium sulfate anhydrous	7487-88-9	Carlo Erba, Italy
Potassium phosphate monobasic	7778-77-0	Carlo Erba, Italy
Creatinine	10028-24-7	Ambeed, USA
Citric acid	77-92-9	neoFroxx, Germany
Yeast extract	8013-01-02	Thermo Scientific, USA
Calcium chloride dihydrate	10035-04-8	Thermo Scientific, USA
Sodium bicarbonate	144-55-8	Thermo Scientific, USA
Iron(II) sulfate heptahydrate	7782-63-0	Thermo Scientific, USA
Sodium chloride	7647-14-5	Thermo Scientific, USA
Ammonium chloride	12125-02-9	Thermo Scientific, USA
Potassium phosphate dibasic	7758-11-04	Thermo Scientific, USA
Sodium sulfate decahydrate	7727-73-3	Thermo Scientific, USA
Bacto™ Peptone		Thermo Scientific, USA
DL-Lactic acid	50-21-5	TCI, Japan
Uric acid	69-93-2	TCI, Japan
