# Supplementary material for: LubriShieldTM—A permanent urinary catheter coating that prevents uropathogen biofilm formation in vitro independent of host protein conditioning
Source: PLoS One. 2025 Jul 10;20(7):e0328167. doi: 10.1371/journal.pone.0328167 (PMC12244716; doi:10.1371/journal.pone.0328167)
Supplement: S3 Table — (PDF) [file pone.0328167.s005.pdf]

S3 Table. Bacterial and fungal strains used in the study

**Strain name**	**Description**
Pseudomonas aeruginosa PA01	Wound isolate *P. aeruginosa*. Common laboratory strain
Staphylococcus aureus B5381	Clinical isolate *S. aureus* (The Department of Microbiology, Karolinska University Hospital, Sweden)
Klebsiella pneumoniae AO15200	Uropathogenic *K. pneumoniae* strain
Escherichia coli CFT073	Uropathogenic *E. coli* strain
Proteus mirabilis CCUG33828	Uropathogenic *P. mirabilis* strain
Enterococcus faecium #11	Clinical isolate *E. faecium* (The Department of Microbiology, Karolinska University Hospital, Sweden)
Enterococcus faecalis #26	Clinical isolate *E. faecalis* (The Department of Microbiology, Karolinska University Hospital, Sweden)
Staphylococcus epidermidis Se19	Isolated from human peritonitis
Candida albicans CCUG70317	Uropathogenic *C. albicans* strain
