# Supplementary material for: LubriShieldTM—A permanent urinary catheter coating that prevents uropathogen biofilm formation in vitro independent of host protein conditioning
Source: PLoS One. 2025 Jul 10;20(7):e0328167. doi: 10.1371/journal.pone.0328167 (PMC12244716; doi:10.1371/journal.pone.0328167)
Supplement: S4 Table — (PDF) [file pone.0328167.s006.pdf]

S4 Table. Fold change in gene expression related to nitrate respiration, arginine fermentation and other hypoxia-related genes in LubriShield™ versus silicone catheter-associated P. aeruginosa

	Gene ID	Gene name	Gene description	Fold change
Nitrate respiration related genes	PA4922	azu	azurin precursor	-1,9
	PA2664	fhp	flavohemoprotein	-4,68
	PA3875	narG	respiratory nitrate reductase alpha chain	-3,24
	PA3874	narH	respiratory nitrate reductase beta chain	-3,72
	PA3872	narI	respiratory nitrate reductase gamma chain	-4,19
	PA3873	narJ	respiratory nitrate reductase delta chain	-3,3
	PA3877	narK1	nitrite extrusion protein 1	-3,74
	PA3876	narK2	nitrite extrusion protein 2	-6,7
	PA3879	narL	two-component response regulator NarL	-2,34
	PA3871	nifM	probable peptidyl-prolyl cis-trans isomerase, PpiC-type	-5,29
	PA0510	nirE	uroporphyrinogen-III C-methyltransferase	-2,85
	PA0516	nirF	heme d1 biosynthesis protein NirF	-2,03
	PA0512	nirH	Siroheme decarboxylase NirH subunit	-1,94
	PA0511	nirJ	heme d1 biosynthesis protein NirJ	-1,91
	PA0518	nirM	cytochrome c-551 precursor	-2,31
	PA0520	nirQ	regulatory protein NirQ	-2,47
	PA0519	nirS	nitrite reductase precursor	-1,7
	PA0524	norB	nitric-oxide reductase subunit B	-2,34
	PA0525	norD	probable dinitrification protein NorD	-3,17
Arginine fermentation related genes	PA5171	arcA	arginine deiminase	-2,57
	PA5172	arcB	ornithine carbamoyltransferase, catabolic	-2,39
	PA5173	arcC	carbamate kinase	-3,07
	PA5170	arcD	arginine/ornithine antiporter	-2,41
	PA0899	aruB	N2-Succinylarginine dihydrolase	-2,57
	PA0901	aruE	N-Succinylglutamate desuccinylase	-1,01
Other hypoxia-related genes	PA5427	adhA	alcohol dehydrogenase	-2,37
	PA2127	cgrA	cupA gene regulator A, CgrA	-2,02
	PA0459	clpC	probable ClpA/B protease ATP binding subunit	-1,26
	PA3126	ibpA	heat-shock protein IbpA	-1,81
	PA4236	katA	catalase	-1,67
	PA0835	pta	phosphate acetyltransferase	-1,23
	PA5495	thrB	homoserine kinase	-2,92
	PA0310		Fe2OG dioxygenase domain-containing protein	-2,08
